# Mimicking Neural Stem Cell Niche by Biocompatible Substrates

**DOI:** 10.1155/2016/1513285

**Published:** 2016-01-06

**Authors:** Citlalli Regalado-Santiago, Enrique Juárez-Aguilar, Juan David Olivares-Hernández, Elisa Tamariz

**Affiliations:** Instituto de Ciencias de la Salud, Universidad Veracruzana, Avenida Luis Castelazo Ayala, s/n, 91190 Xalapa, VER, Mexico

## Abstract

Neural stem cells (NSCs) participate in the maintenance, repair, and regeneration of the central nervous system. During development, the primary NSCs are distributed along the ventricular zone of the neural tube, while, in adults, NSCs are mainly restricted to the subependymal layer of the subventricular zone of the lateral ventricles and the subgranular zone of the dentate gyrus in the hippocampus. The circumscribed areas where the NSCs are located contain the secreted proteins and extracellular matrix components that conform their niche. The interplay among the niche elements and NSCs determines the balance between stemness and differentiation, quiescence, and proliferation. The understanding of niche characteristics and how they regulate NSCs activity is critical to building* in vitro* models that include the relevant components of the* in vivo* niche and to developing neuroregenerative approaches that consider the extracellular environment of NSCs. This review aims to examine both the current knowledge on neurogenic niche and how it is being used to develop biocompatible substrates for the* in vitro* and* in vivo* mimicking of extracellular NSCs conditions.

## 1. Introduction

Stem cells are characterized by their extensive potential for proliferation and differentiation, as well as their major role in homeostasis and tissue regeneration. Although stem cells are a promising source for cell replacement therapies and cell regeneration after injury or disease, their use is still limited because there are several factors that must be taken into account, such as survival, tissue integration, specific differentiation, and functionality. In order for them to be considered within regenerative medicine, it is imperative to understand their* in vivo* biology and microenvironment, or niche. In recent years, the use of* in vitro* models that simulate various components of the niche has helped the understanding of the role of the various factors that compose it and even the design of artificial models that recapitulate microenvironment conditions [1, 2]. In that sense, biocompatible substrates are an alternative for the incorporation of different physical and chemical properties that can modulate the biology of stem cells and improve their manipulation [3]. This paper will review some of the main extrinsic characteristics of the neurogenic niche and how current knowledge about it is being used to design biocompatible substrates that mimic the microenvironment of neural stem cells in order to regulate their biology, as well as the impact this may have on the future of tissue regeneration therapies.

## 2. Embryonic and Adult Neural Stem Cells

Neural stem cells (NSCs) originate the main cell types in the central nervous system (CNS) during development and adulthood. These cells are able to self-renew through cell division and have the capacity to generate specialized cell types. NSCs generate other NSCs, which maintain their differentiation potential and their proliferation or self-renewal capacity, and/or originate transit-amplifying cells or neural progenitor cells (NPCs), which display decreased proliferative potential and limited capacity to differentiate into neurons, astrocytes, and oligodendrocytes. From early embryonic development up to early postnatal stages, neurons are the main cell types generated, while late embryogenesis is characterized by the production of both astrocytes and oligodendrocytes, which continues during postnatal stages and throughout adult life [4]. The process of generating functional neurons or glial cells from precursors is defined as neurogenesis and was thought to occur only during the embryonic and perinatal stages in mammals. Currently, it is widely accepted that neurogenesis takes place in the adult brain and that the neural stem cells of this organ are descendants of their embryonic counterparts.

A number of significant questions remain regarding the biology of embryonic and adult neural stem cells. How is the fate of NSCs determined? What determines whether NSCs remain in their stem stage or differentiate into one of the three mature phenotypes? Over the last few years, it has become clear that NSCs are sensitive to multiple signals during development, including extracellular matrix proteins, growth and transcription factors, or even the interaction with different cell types in their proximity [5, 6]. Although apparently of the same nature as their embryonic counterparts, adult NSCs show different responses to the same regulators. At the same time, these cells are mostly quiescent in the adult brain with a low neuron production rate in contrast to the high proliferative rate of the embryonic NSCs. Additionally, neuronal maturation is accomplished at a slower rate in the adult brain than in the embryo. Although the reason for these differences is not clear, it has been reported that the acceleration of the maturation rate sometimes leads to the aberrant integration of newborn neurons in the adult hippocampus [7]. It has been suggested that, besides intrinsic differences, changes in the microenvironment surrounding neural stem cells during both development and adult life modulate their biological response [7].

**Figure 1:**
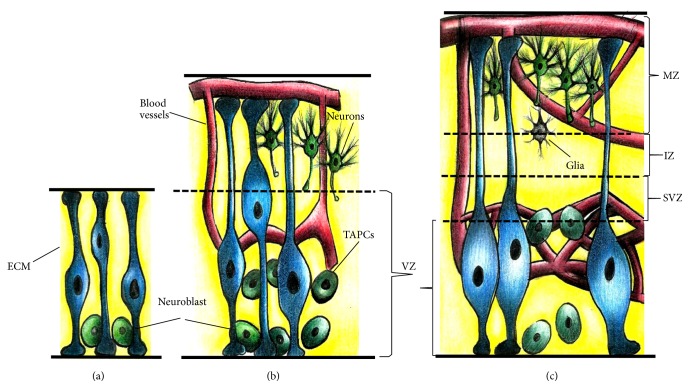
Neural stem cell niche in the early stages of CNS development. (a) During early embryogenesis, neuroepithelial cells transform into radial glial cells (RGCs) with cell process elongating toward the pial surface of the neural tube. RGCs divide asymmetrically to form neuroblasts. (b) Neuroblast division generates progenitors known as “transit-amplifying progenitor cells” (TAPCs), which divide rapidly and generate the first local neural niche or ventricular zone (VZ); at this stage the first blood vessels invade the neural tube from the dorsal region toward the VZ zone and extend their branches tangentially to the pial surface. (c) In the forebrain, TAPC proliferation produces a second germinal zone, the subventricular zone (SVZ). In this zone, postmitotic neuroblasts and glioblasts migrate toward the dorsal intermedia and marginal zones and produce neurons and glial cells. CNS, central nervous system; ECM, extracellular matrix; IZ, intermediate zone; MZ, marginal zone.

During early embryogenesis, NSCs are not specifically localized and are instead organized as a single layer of proliferating neuroepithelial cells in the neural tube. Early in the neural tube formation, cells at the junction of the tube form the neural crest cells, which migrate out of the tube to form the neurons and glia of the peripheral nervous system as well as other non-nervous system cells, such as melanocytes, chondrocytes, and craniofacial osteocytes [86]. Neuroepithelial cells in the neural tube divide symmetrically, generating two identical daughter cells. Once this population has increased, they switch to a new form of asymmetrical division, producing two distinct daughter cells, the typical self-renewing stem cell and the neuroblast, with the former transforming into radial glial cells (RGCs) that exhibit neuroepithelial and glial proteins, extending a long cell process towards the outer neural tube region or pial surface (Figure 1(a)). As brain development proceeds, the proliferation of RGCs and neuroblasts generate several layers that surround the interior face of the neural tube, leading to the first local neural niche called the ventricular zone (VZ) (Figure 1(b)). The neuroblasts and glioblasts with high proliferating capacity also termed intermediate progenitor or “transit-amplifying progenitor cells” (TAPCs) generate postmitotic cells that finally differentiate into neurons and glial cells. In the forebrain region, the TAPCs accumulate above the VZ, forming a second germinal zone, the subventricular zone (SVZ) (Figure 1(c)). All these populations are also in close contact with cells of nonneural origin, such as endothelial cells from the blood vessels, microglia, and pericytes [5, 87]. Interactions among all these cells, together with the temporal and spatial synthesis of soluble and insoluble factors during CNS development, result in the establishment of the intricate neural network that will support the function of this system in postnatal life [88].

**Figure 2:**
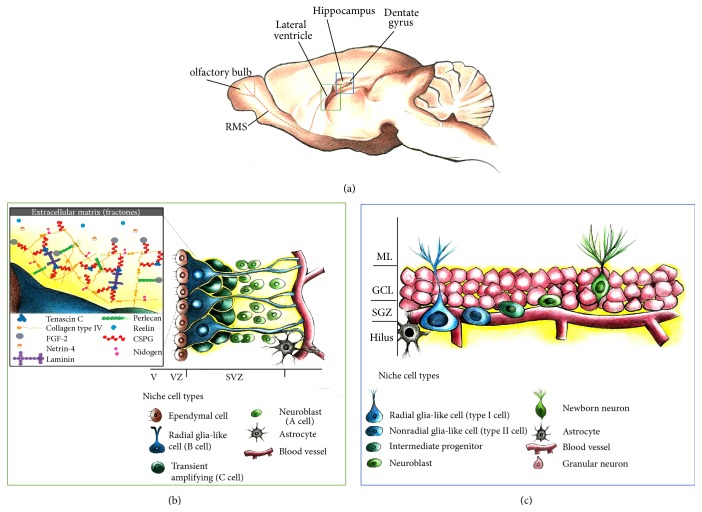
Neural stem cell niche in the adult dentate gyrus and subventricular zone. (a) Sagittal section view of an adult rodent brain showing the two main restricted regions where active adult neurogenesis is present, the dentate gyrus in the hippocampal formation and the lateral ventricle, from which type A cells migrate to form the rostral migratory stream (RMS) toward the olfactory bulb. (b) Neural stem cell niche in the subventricular zone (SVZ). Three types of progenitor cells are found close to the ependymal cell layer in the SVZ: a population of radial glia-like cells (type B cells) have the potential to serve as adult neural stem cells (NSCs) and generate transit-amplifying nonradial NSCs (type C cells), which later give rise to neuroblasts (type A cells). The SVZ includes several ECM components (yellow), called fractones (inset), which make contact with all the cell types, including the blood vessels and astrocytes in this region. (c) In the adult subgranular zone (SGZ), a population of radial glia-like cells (type 1 cells), along with nonradial glia-like cells (type 2 cells), generate neuroblasts. These neuroblasts then migrate into the granule cell layer and mature into neurons. CSPG, chondroitin sulfate proteoglycan; FGF2, fibroblast growth factor 2; GCL, granular cell layer; ML, molecular layer.

After the embryonic phase of CNS development, some NSCs populations remain in specific neurogenic niches throughout the lifespan of the brain. Two specific and well-described neurogenic regions remain in the adult brain after the embryonic phase, the subgranular zone (SGZ) in the dentate gyrus (DG) of the hippocampus and the subventricular zone (SVZ) of the lateral ventricles (Figure 2). While the neurogenesis in these neurogenic sites results in the generation of new neurons in the brain, there are differences in the type of neurons generated. Neurogenesis produces dentate granule cells in the SGZ of the DG of the hippocampus, while neurogenesis in the SVZ of the lateral ventricles produces interneurons that migrate to the olfactory bulb (Figure 2(a)). The production of mature neurons in these neurogenic sites comprises several steps that resemble embryonic neurogenesis.

Neurogenesis of the adult SVZ begins with the activation of quiescent radial glia-like cells (termed type B cells) in the subventricular zone in the lateral ventricle and continues with the proliferation of transit-amplifying progenitor cells (type C cells), resulting in an increase in the neuroblast population (type A cells) or glia (oligodendrocytes or astrocytes) (Figures 2(a) and 2(b)). In the rostral migratory stream (RMS), type A cells form a chain and migrate toward the olfactory bulb through a tube formed by astrocytes. Upon reaching the olfactory bulb, immature neurons leave the RMS and migrate radially toward the glomeruli, where they differentiate into different subtypes of interneurons that, finally, are synaptically integrated [89] (Figure 2(a)). Type B cells are in close contact with the ependymal cell layer through a thin apical process and with SVZ vasculature through a basal process. This structural polarity allows type B cells to be in simultaneous contact with both vascular and cerebrospinal fluid (CSF) compartments [7] (Figure 2(b)). In other cases, hippocampal neurogenesis in the DG begins, as in SVZ neurogenesis, with the proliferation of radial (type I cell) and nonradial (type II cell) precursors that give rise to intermediate progenitors, which in turn generate neuroblasts (Figures 2(a) and 2(c)). Unlike in SVZ neurogenesis, immature neurons are not required to migrate long distances in order to initiate the differentiation process. The new immature neurons move into the inner granule cell layer and differentiate into dentate granule cells in the hippocampus. Within days, newborn neurons extend dendrites toward the molecular layer and project axons through the hilus toward the CA3. New neurons follow a stereotypic process for synaptic integration into the existing circuitry [89].

Besides the classic neurogenic sites, there is evidence indicating the presence of other neurogenic sites in the brain that are more evident after injury or growth factor stimulation, such as the walls of the third and fourth ventricle and the circumventricular organs. All of these sites are close to blood vessels [90], although their detailed characterization and contribution after brain damage are still the subject of intense study.

## 3. Neural Stem Cell Niche: Characteristics and Relevance

The anatomical distribution of many stem cells has been a troublesome task due to the low accessibility and restricted areas where they are located. Currently it is accepted that the tissue areas where stem cells lie are specialized microenvironments with specific cellular, chemical, and physical properties [2].

As mentioned above, embryonic and adult NSCs are influenced by their microenvironment. The microenvironment concept is related to the presence of extracellular matrix proteins and soluble factors such as hormones and growth factors in the extracellular space (some of which are summarized in Table 1). However, these are not the only factors that can influence the biology of the NSCs. It has been evident that interactions with neighboring cells are also a relevant modulator of the biology of these cells in both the embryo and the adult. Endothelial cells, astrocytes, ependymal cells, microglia, mature neurons, and the progeny of adult neural stem cells are additional regulators of the fate of the NSCs. All of these elements in the cellular microenvironment constitute the neurogenic niche that anatomically houses stem cells and functionally controls their development* in vivo* [90]. As excellent and extensive reviews have been published on the neurogenic niche [5, 88, 91–94], this section is only a brief summary, which includes examples of the different factors that could be taken into account in biocompatible substrate design.

The neurogenic niche determines whether a NSCs divides or remains as a quiescent cell as well as whether it survives, dies, proliferates, migrates, or differentiates into different neural cells [6, 95, 96]. During the development of the CNS, the VZ of the neural tube is mainly comprised by the proliferative cells of the neuroepithelium. Segmentation and regionalization of the neural tube modify and restrict the neurogenic areas during the developmental stages, leading to a niche that is spatially, chemically, and cellularly variable [97, 98]. During the changes, stem cells and progenitors in the VZ and SVZ remain in contact with specific extracellular components such as growth factors, ECM components, and cells that modulate their division and differentiation. During the development of the neocortex, for example, neuroepithelial cells proliferate to form several layers of cells surrounding the lumen of the neural tube. As mentioned above, the inner cells transform into RGC, whose asymmetric divisions generate self-renewing cells that stay in the VZ, and progenitor cells that migrate to the SVZ. After several divisions, the progenitors go from the SVZ to their destination through radial and tangential migration and differentiate into neurons and glia [5, 96, 99]. Several transcription factors, proteins related to cell polarity, such as cadherins and nectin, as well as signaling components are all activated in a complex that promotes the dissolution of cell adherens junctions and the reorganization of the actin cytoskeleton to favor progenitor migration [100].

The vascularization of the neural tube is closely related to neural development. The timing of angiogenesis is similar to the neurogenesis. The RGCs secrete several growth factors, such as vascular endothelial growth factor (VEGF), transforming growth factor beta two (TGF*β*-2), and fibroblast growth factor two (FGF2), which, in turn, induce vasculature development, while the growth factors secreted by endothelial cells, such as VEGF and Jagged-1 (a Notch ligand), influence neurogenesis [5]. Furthermore, nonneural cells are also involved in establishing and supporting the neurogenic niche [5]. For example, it has been shown that, during brain development, pericyte cells synthesize the sonic hedgehog (Shh) protein, which plays an important role in the proliferation of the neuroepithelial cells that conform the VZ [101].

In the postnatal and adult brain, the main neurogenic niches in the SVZ and the DG play a role in maintaining the balance between stemness and NSCs differentiation. The vasculature also emerges as an important and integral component in the adult stem cell niches of both the hippocampus and SVZ [102, 103]. There is increasing evidence showing a dense network of blood vessels in the hippocampus that spans beneath the RGCs and dorsal to the SVZ in the lateral ventricles. This network is closely associated with the NSCs (including the TAPCs) and has long processes oriented along the neuroblast chain and within the microglia cells [104]. It has been shown that vasculature interactions promote the neuroproliferation and neuroprotection of the NSCs and the migration of astroglial cells through the secretion of regulatory factors in an autocrine or paracrine manner [5, 105]. Transcriptome analysis of endothelial cells of the CNS shows the presence of several factors involved in neurogenic niche [106]. VEGF and TGFbeta1 are synthesized and secreted by endothelial vascular cells in the SVZ [36] and VEGF is also secreted by NSCs in the hippocampal neurogenic niche [107].

A great variety of factors active in the NSCs niche modulate the components of this site, with some of them preventing terminal differentiation and preserving the NSCs pool. A highly conserved secreted protein with a determinant role during the dorsoventral patterning of the neural tube, Shh, is an example of such a factor. Its mutation during embryo development leads to a reduction of telencephalon and diencephalon [108] and its ectopic expression increases the generation of oligodendrocytes [109]. On the other hand, Shh is involved in maintaining the pool of progenitor cells in the postnatal brain [110].

Another factor, Notch1, is a transmembrane protein of the Notch family of proteins playing a variety of roles during development. Notch1 induces the expression of transcriptional repressor genes such as* Hes1*, leading to the repression of proneural gene expression and the maintenance of the NSCs [105]. The importance of the Notch signaling requirement has been shown in inducible knockout mice where stem cell self-renewal and expansion are disrupted leading to neural stem cells depletion [105]. Bone morphogenetic proteins (BMPs), members of the TGF*β* superfamily first identified by their role in bone induction [111], and the highly conserved Wnt proteins are all secreted proteins identified as morphogens due to their concentration dependent role during development. However, they also play a critical role in maintaining adult NSCs niches, activating the proliferation of type B astrocytes, the transit-amplifying type C cells in the SVZ, and neurogenesis in the SVZ [7].

The basal lamina and ECM provide both structural shape and mechanical support for the developing and adult nervous systems [93]. These components of the neurogenic niche act as a scaffold for the incorporation of a variety of ECM molecules and growth factors. Some of the most important ECMs that play a role in the regulation of the biology of the NSCs and progenitors cells are laminin, collagen IV, nidogen, perlecan, the glycoprotein tenascin C, the chondroitin sulfate proteoglycans (CSPG), and the heparan sulfate proteoglycans (HSPG) [112].

Laminin is an ECM heterotrimeric protein located in the lateral ventricular wall of the SVZ. In the mammalian brain and spinal cord, the basal lamina in the neurogenic site forms branches, or “finger-like processes,” called fractones, that extend from the ependymal cells and blood vessels. Laminin and its receptor *α*6*β*1 integrin have been detected in these structures in the NSCs of the SVZ that lie near the vascular cells [87]. Additionally, heparan sulfate, collagen IV, nidogen, and perlecan have been described as components of the fractones that make contact with the transit-amplifying cells, suggesting that, in combination with growth factors, these structures have a role in adult neurogenesis. Specifically, HSPG, CSPG, and perlecan bind to growth factors such as FGF-2, a potent mitogenic factor for the NSCs, suggesting that these ECM components promote growth factor activity in the NSCs niche [113].

Secreted by type B cells, the astrocytes surrounding migratory type A cells, and the RGC in embryos, tenascin C is a major component of the ECM in the adult SVZ [114]. Tenascin C regulates the expression of EGF receptors in the embryonic NSCs and has been reported to alter cell response to mitogenic growth factors by enhancing sensitivity to FGF-2 and promoting EGF acquisition [115, 116]. Tenascin C also regulates oligodendrocyte precursor proliferation, while the isoform tenascin R induces the maturation of oligodendrocyte precursors [114].

Reelin is a large ECM glycoprotein that plays an important role not only in neuronal migration during cortex development [117] but also in the NSC niche, where it is expressed in the SGZ in adult hippocampus and regulates NSCs maintenance and migration [82, 118].

Proteins that act as neuronal guidance factors are also associated with the regulation of the adult neurogenic niche. The Eph/ephrin receptor-ligand complex is large class of membrane associated receptors and ligands that are involved in axon guidance [119] and mediate the cell-to-cell signaling that promotes the proliferation of the NPC in the SVZ [119, 120]. Netrins are a laminin-related family of proteins that act as a guidance cue for neuronal projection and have a role in inducing the migration of NSCs during cerebellar development [121]. Recently, Netrin-4 was found to interact with components of the ECM in a complex that is able to control the proliferation of the adult NSCs and their migration to the mouse olfactory bulb [122].

Altogether, this evidence suggests that ECM components and soluble proteins regulate the biology of the NSCs in the neurogenic niche.

## 4. Biocompatible Substrates for Mimicking the Neural Stem Cell Niche

The growing body of evidence supporting the influence of the extracellular environment on stem cells raises the question as to whether* in vitro* culture conditions have the optimal characteristics for growing stem cells outside the body. Evidence is beginning to show that constructing* in vitro* microenvironments that incorporate some of the niche elements where stem cells lie* in vivo* could be advantageous to the understanding of stem cell biology and possible applications in regenerative medicine [2, 90, 123–125].

Evidence has shown that extracellular environmental characteristics such as protein composition, protein anchoring density, stiffness, and topography are important parameters to consider [2, 126]. Polymeric biomaterials can be designed and modified to obtain compatibility characteristics with cells and tissues and to provide substrates, cells, and proteins. Compatible materials can be functionalized to provide bioactive proteins and peptides that signal cells to attach, proliferate, or differentiate and can modulate physical characteristics such as stiffness or topography, even at a nanometric scale [93, 127].

There are two main approaches for the use of biomaterials: as delivery vectors for proteins and growth factors with important effects in stem cell biology and as scaffolds for the manipulation of cell characteristics or for improving viability. More recent studies are using both approaches to design more accurate scaffolds or substrates, such as bioactive polymers with multivalent ligands, or 3D substrates with several crosslinking densities that are functionalized using active peptides [3, 93, 128, 129].

## 5. Bioactive Factors Coupled to Compatible Substrates

As mentioned before,* in vivo* growth factors are coupled to the ECM of the neurogenic niche, thus forming site specific regions of active factors. Growth factors are coupled by electrostatic interactions with ECM proteins such as glycosaminoglycans, which are one of the most abundant components of the ECM, thus regulating growth factor accessibility to cells [130].

Polymeric materials are usually inert and do not have chemical interactions with either cells or proteins, a property which improves biocompatibility in that it impairs the adsorption of nonspecific proteins, avoiding the recognition by innate immunity system [131, 132]. However, bioactive peptides or proteins can be coupled to polymeric materials to support cell adhesion, viability, stemness, or differentiation and can be used as either delivery vectors or scaffolds through the coupling of specific cell adhesion proteins or peptides in 2D or 3D cultures [123, 128, 133–135].

The use of biomaterials for the delivery of growth factors offers the possibility of controlling the place and rate of delivery and avoiding unspecific or pleiotropic effects. An important issue to consider is whether to deliver growth factors by releasing them or coupling them to the polymer. It has been shown that EGF, covalently attached to a substrate, leads to a greater expansion of human NPC as compared to soluble EGF [136], while platelet derived growth factor (PDGF), coupled to an agarose hydrogel, induces NPC differentiation into oligodendrocytes. However, the degree of the expression of myelin oligodendrocyte glycoprotein (MOG) is higher when the NPCs are exposed to soluble PDGF [137]. Covalently linked growth factors cannot be internalized by cells, meaning that their activity and function could be disrupted. Some actively released scaffolds are being developed in response to these problems, such as the heparin functionalized poly(ethylene glycol) (PEG) hydrogels [138, 139] and fibrin gels with growth factors coupled via heparin binding [140]. In this latter approach, the simultaneous release of neurotrophin-3 (NT-3) and PDGF improved neural induction and decreased astrocyte differentiation, indicating that a biomimetic scaffold could be designed to use several growth factors with specific kinetic release and dosages [129].

The potential of biomimetic scaffolds that provide and expose cells to growth factors for* in vivo* application has been recently shown using hyaluronic acid gels functionalized with ephrin B2 and Shh. Interestingly, a multivalent polymer was designed to cluster ephrin receptors which substantially increased the quantity of new neurons formed in an adult neurogenic zone, such as the hippocampus, and in nonneurogenic zones, such as the cortex and striatum. Neurogenic activity was also induced in geriatric rodents, with decreased neurogenesis in the hippocampal region [3]. Although there remain many aspects to consider before the clinical application of this strategy, it is evident that the characterization of neurogenic niches and their application in compatible bioactive materials could be a promising approach.

Cell-material interaction is crucial for the modulation of cell behavior. Polymeric materials can also be functionalized to expose peptides, adhesion molecules, or chemical groups that mimic those in cells and the ECM and exert their function through specific ligand-receptor recognition, or electrostatic interaction. The Arg-Gly-Asp (RGD) tripeptide motif present in ECM components, such as laminin, can modify adult hippocampal NSCs proliferation and differentiation [126, 141], with –SO_3_H exposed groups favoring the differentiation of embryonic NSCs into oligodendrocytes, while –NH_2_ exposed groups induce neuronal differentiation [142]. Functionalized polymers can be used as scaffolds to improve NSCs transplantation and thus support their survival and integration into brain tissue, especially when large tissue deficits are present, such as after traumatic brain injury, cerebral ischemia, or a transected spinal cord [1, 143, 144].

## 6. Stiffness, Topography, and Neural Stem Cells

The physical properties of the extracellular environment also influence stem cell behavior. Stiffness is defined as the resistance of a material to deformation when force is applied and is mainly related to material composition and structure, while topography refers to the tridimensional shape and relief of a material, in this case at micro- or nanoscale.

Evidence has shown that physical properties can modify cell behavior and modulate stem cell differentiation capabilities by modulating gene expression, integrin clustering, the formation of cell adhesion, and cytoskeleton regulation [8, 145–147].

Although brain stiffness has been difficult to measure and despite the variation in the data reported according to the technique used, it has been accepted that the brain is one of the softest tissues of the body. Brain stiffness can change according to age and the area of the brain. The adult brain is stiffer than the juvenile brain (~0.040 kPa in postnatal 10 rat brain samples versus ~1.2 kPa in adult rat brain samples). Interestingly, most of the cortical subregions are stiffer than the dentate gyrus and CA1 regions in the hippocampus [148]. These differences are related to the water, protein, and lipid content. Water content significantly decreases with age, while lipid and protein content increase [148]. Another important factor is the composition of the ECM, as sulfated glycosaminoglycan increases with age [97]. In a developing brain, there are also important changes in their mechanical properties, with, for example, a gradual increase in stiffness in the VZ and SVZ being closely related to neurogenic stage, neuron maturation, and ECM composition changes during development [97, 149].

Previous studies have demonstrated that mechanical properties of the substrate, in conjunction with cell adhesion ligands, can preserve the undifferentiated state of human embryonic stem cells [150] or induce differentiation in several cell phenotypes depending on the degree of stiffness [145]. In the case of neural stem cells, Leipzig and Shoichet (2009) have shown that softer substrates comprising photo cross linkable methacrylamide chitosan (MAC) hydrogels functionalized with laminin induce higher proliferation and neuronal differentiation levels for the NPCs obtained from the SVZ region of the forebrains of adult rats. In contrast, the proliferation rate decreases for the NPCs in stiffer gels, which differentiate preferably into oligodendrocytes [151]. Similarly, studies using NPCs from adult rat hippocampi showed higher proliferation rates for the NPCs in hydrogels of ~0.1 to ~0.5 kPa than in softer substrates (~0.01 kPa), reaching a proliferation peak at 1 to 4 kPa. In addition, the NPCs preferentially differentiate into the neural phenotype in soft substrates (~0.1–0.5 kPa), while glial phenotypes are predominant in stiffer substrates (~1–10 kPa) [152]. Notably, the influence of soft substrates in neuronal differentiation is maintained in 3D cultures when the NPCs from adult hippocampi are grown in alginate hydrogels [153].

An interesting fact is that NPCs reach high proliferation and neuronal differentiation levels in substrates with low stiffness that are similar to those reported for brain tissue. However, the influence of soft gel substrates on embryonic NPC differentiation seems to be different, in that it has been shown that glial differentiation is enhanced in soft polydimethylsiloxane gels (PDMS), while neuronal differentiation is not affected by soft gels, as previously described [154]. These differences could be attributed to the origin or stage of development of the NPCs, or even the characteristics of the substrate, as reported previously by Trappmann et al. (2012) [147].

The effects of substrate stiffness can be mediated by the modulation of gene expression, as reported earlier using mesenchymal stem cells (MSCs). When MSCs are grown for longer periods of time on stiffer substrates, the proosteogenic genes are expressed in a nonreversible way. However, when they are grown for short periods of time on the same substrate, the MCSs are still able to reverse the expression of proosteogenic genes and begin to express neurogenic genes, which shows evidence of a sort of mechanical memory that could have important implications for the way stem cells are being grown and expanded* in vitro* [8].

The micro- and nanoscale topography of a substrate have been shown to be another important factor for the manipulation of stem cell behavior. Topography can be altered, using pillars, grooves, pits, or fibers to modify cell orientation and cytoskeleton arrangement. The microscale distribution of cell adhesion points and, therefore, the manipulation of cell shape has been shown to be crucial to the direction of MSC differentiation toward osteogenic or adipogenic lineage. These effects are mediated by actin-myosin tension [155]. Fiber diameter can also influence NSCs differentiation; laminin-coated electrospun polyethersulfone (PES) fiber meshes of 283 nm increased oligodendrocyte differentiation by 40%, while 749 nm fibers increased neuronal differentiation by 20% as compared with culture plates [156]. Aligned fiber substrates of 480 nm upregulate neuronal differentiation through the induction of Wnt/*β* catenin signaling, which is a crucial pathway during neurogenesis in embryos and adults and is more favorable to the survival of neuronal cells as compared to the oligodendrocytes [124]. It has also been shown that micropatterned substrates with aligned microgrooves functionalized with laminin align the direction of hippocampal NSCs growth and facilitate their differentiation into neurons when they are cocultured with astrocytes [157].

Although the multiple approaches to the physical and chemical manipulation of biocompatible substrates show their capability to manipulate NSCs behavior, there are still several characteristics that must be considered, such as the complexity of the interrelation of multiple signals and how these can affect NSCs behavior depending on their origin and the intrinsic stem cell characteristics.

## 7. Conclusions

The study of the numerous elements present in the neurogenic niche and how they interact with stem cell behavior contributes to understanding the importance of extrinsic signals for NSCs destiny. This knowledge is being used to mimic the neurogenic niche for* in vitro* and* in vivo* applications. Although the results obtained up to now show promise, a more accurate biomimetic substrate for* in vitro* studies and regenerative medicine is still a long way off. Several factors must be taken into account, such as the soluble factors and ECM components present in the niche and the physical properties of the substrate. The type and the origin of the stem cells intended to provide a niche must also be considered. The ideal biomimetic scaffold should, therefore, incorporate some of the main factors that control stem cell behavior. Multidisciplinary approaches to developing the most accurate niche-like substrates and to understanding their biological implications are a fascinating field that will help develop stem cells knowledge.

## Figures and Tables

**Table 1:** Soluble factors and ECM components associated with neurogenic niches.

Factors	Location	
	Embryonic niche	Adult niche
*Growth factors*		
BDNF (brain derived neurotrophic factor)	Forebrain (induces proliferation of embryonic NSC) [8]	DG, SVZ (promotes neurogenesis) [9, 10]

Cystatin-C	Neocortex (promotes astrogenesis and suppresses oligodendrogenesis) [11]	DG, blood vessels (promotes neurogenesis) [12]

FGF-2 also known as basic FGF (fibroblast growth factor)	Neuroepithelial cells and RGC (essential for self-renewal and maintaining multipotency) [5, 13]	SVZ, SGZ of DG (regulation of NSC and progenitor cells) [14]

IGFs (insulin growth factors)	Choroid plexus epithelium, CSF, striatal primordial, and neocortex (stimulates survival and proliferation of NSC) [14–18]	CSF, DG, and hypothalamus (regulation of NSC proliferation) [19, 20]

EGF (epidermal growth factor)	Striatal primordial (induces proliferation of NSC) [21, 22]	SVZ (induces proliferation of the NSC) [23]

PDGF-A, PDGF-B (platelet derived growth factor-A, platelet derived growth factor-B)	SVZ (stimulates differentiation into astrocytes and oligodendrocytes) [24, 25]	SVZ (induces differentiation of the PDGF-responsive progenitors into glia cells) [26]

PEDF (pigment epithelium derived factor)	ND	SVZ (promotes stemness of the NSC) [27, 28]

GDF-11 (growth differentiation factor-11)	Neuroectodermal tissue (involved in the earlier steps of the neural plate patterning) [29, 30]	ND

GDF-15 (growth differentiation factor-15)	Hippocampus (regulates the migration and differentiation of hippocampal precursors by promoting EGFR signaling) [31]	ND

GDNF (glial cell line derived neurotrophic factor)	Ventral mesencephalon (neurotrophic factor for NSC at nigrostriatal region) [32]	DG (promotes astrogliogenesis from NPC) [33, 34]

TGF-*β*1 (transforming growth factor-*β*1)	Neocortex (regulates differentiation into astrocytes) [35]	SGZ, GCL of the hippocampus, and EC (promotes stem cell quiescence and the survival of newly generated neurons)

VEGF (vascular endothelial growth factor)	Ventricular neuroectoderm, RGC, and EC (angiogenic and mitogenic factor)	SVZ, SGZ of DG, and EC (stimulates neurogenesis) [36–38]

CNTF (ciliary neurotrophic factor)	Choroid plexus, VZ, and forebrain germinal zone (expansion and self-renewal of NSC) [39, 40]	SVZ, SGZ of the DG (regulates the balance between NSC self-renewal and the generation of neuronal progenitors) [41, 42]

LIF (leukemia inhibitory factor)	Choroid plexus and VZ (expansion and self-renewal of NSC) [39]	SVZ, CA3 of the hippocampus (self-renewal of NSC and proliferation of oligodendrocyte progenitor cells) [43, 44]

*Hormones*		
GH (growth hormone)	Striatal primordial, neocortex (stimulates survival of the NPC and proliferation and differentiation of cortical cells) [45, 46]	SVZ (regulates proliferation of NPC) [47, 48]

Ghrelin	Spinal cord (induces proliferation of NSC) [49]	SVZ, SGZ of the hippocampus (regulates proliferation and differentiation of progenitor cells and migration of neuroblasts in the SVZ) [50]

EPO (erythropoietin)	Ganglionic eminences (promotes production of neuronal progenitors) [51]	SVZ, SGZ of the hippocampus (regulates proliferation and differentiation of progenitor cells and the migration of neuroblasts in the SVZ) [51]

*Morphogens*		
BMPs (bone morphogenetic proteins)	Dorsal midline of the telencephalon and cortical hem (develops mouse olfactory system and SVZ and modulates response to EGF) [52–54]	SVZ, hippocampus (promotes the survival of SVZ-derived neurons and maintenance of the quiescent state of NSC) [55, 56]

SHH (sonic hedgehog)	Dorsal telencephalon, CSF, pericytes (dorsoventral patterning, development of cortex and hippocampus)	SVZ and SGZ of DG (chemotaxis and maintenance of the NSC) [57, 58]

Wnt	Caudomedial cortex (expansion of caudomedial cortical progenitor cells) [59]	Hippocampus (regulator of neurogenesis) [60]

Notch-1	Developing telencephalon (maintenance of the telencephalic NSC) [3, 61]	DG and SVZ (modulates adult neurogenesis) [62–64]

FGF-8a, FGF-8b (fibroblast growth factor-8a, fibroblast growth factor-8b)	Mid-hindbrain (mitogen for NSC) [65]	ND

Retinoic acid	Choroid plexus, CSF, and ganglionic eminence (migration of NPC to cerebral cortex and regulation of neuronal differentiation) [66]	Infrapyramidal and suprapyramidal layers of the hippocampus, SGZ, and SVZ (regulates proliferation of NPC in the SGZ and neuronal differentiation) [66, 67]

*Extracellular matrix components*		
HSPG (heparan sulfate proteoglycan)	Neuroepithelial cells (essential for many of the protein effectors involved in pluripotency and neural differentiation) [68]	SVZ, fractones [69]

CSPG (chondroitin sulfate proteoglycan)	Neocortex and ganglionic eminence (essential for mitogens that promote self-renewal and neurogenesis) [70, 71]	SVZ (essential for mitogens involved in NSC maintenance) [70, 72]

Heparin	Neocortex (interaction with growth factors, proliferation of NSC) [73]	DG, SVZ (essential for mitogens involved in neurogenesis) [74]

Laminins	Neuroepithelial cells, VZ (promote formation of properly polarized cortical neuroepithelium) [75]	SVZ, fractones (enhance NSC proliferation and self-renewal) [69, 76]

Collagen	Neocortex (collagen IV regulates corticogenesis by inhibiting cell proliferation and glial cell differentiation and promotes neuronal differentiation) [77]	SVZ, fractones (collagen I in subependymal layer and the basal lamina of blood vessels) [69]

Vitronectin	Developing spinal cord (promotes differentiation into oligodendrocytes) [70]	ND

Tenascin	Neocortex, RGC, and spinal cord (tenascin C promotes EGF response of NSC and promotes oligodendrocyte precursor proliferation and astrocytic lineages) [71, 72]	SVZ and olfactory bulb (tenascin R regulates neurogenesis and radial migration in the olfactory bulb) [78, 79]

Reelin	Marginal zone of developing cortex (controls differentiation, migration, and proliferation of NSC) [80, 81]	SGZ, cortex (regulation of quiescence and migration of NSC and maintenance of cortical architecture) [82, 83]

Perlecan	Ventral forebrain and neocortex (maintains the basal lamina and influences the size of ventral and cortical telencephalic structures) [84]	SVZ, fractones (promotes growth factor activity in the NSC niche) [85]

NSC, neural stem cell; SVZ, subventricular zone; VZ, ventricular zone; RGC, radial glial cell; SGZ, subgranular zone; CSF, cerebrospinal fluid; NPC, neural progenitor cell; GCL, granular cell layer; DG, dentate gyrus; EC, endothelial cell.
